# Repositioning of antiarrhythmics for prostate cancer treatment: a novel strategy to reprogram cancer-associated fibroblasts towards a tumor-suppressive phenotype

**DOI:** 10.1186/s13046-024-03081-0

**Published:** 2024-06-11

**Authors:** Valentina Doldi, Monica Tortoreto, Maurizio Colecchia, Massimo Maffezzini, Stefano Percio, Francesca Giammello, Federico Brandalise, Paolo Gandellini, Nadia Zaffaroni

**Affiliations:** 1 Molecular Pharmacology Unit, Department of Experimental Oncology, Fondazione IRCSS Istituto Nazionale Dei Tumori, Milan, 20133 Italy; 2 Vita-Salute San Raffaele University, IRCCS San Raffaele Hospital and Scientific Institute, Milan, 20132 Italy; 3 Department of Urology, Hospitals of Legnano and Magenta, Milan, 20013 Italy; 4 Department of Biosciences, University of Milan, Milan, 20133 Italy

**Keywords:** Cancer-associated fibroblasts, Drug-repositioning, Antiarrhythmics, Prostate cancer

## Abstract

**Background:**

Cancer-associated fibroblasts (CAFs) play a significant role in fueling prostate cancer (PCa) progression by interacting with tumor cells. A previous gene expression analysis revealed that CAFs up-regulate genes coding for voltage-gated cation channels, as compared to normal prostate fibroblasts (NPFs). In this study, we explored the impact of antiarrhythmic drugs, known cation channel inhibitors, on the activated state of CAFs and their interaction with PCa cells.

**Methods:**

The effect of antiarrhythmic treatment on CAF activated phenotype was assessed in terms of cell morphology and fibroblast activation markers. CAF contractility and migration were evaluated by 3D gel collagen contraction and scratch assays, respectively. The ability of antiarrhythmics to impair CAF-PCa cell interplay was investigated in CAF-PCa cell co-cultures by assessing tumor cell growth and expression of epithelial-to-mesenchymal transition (EMT) markers. The effect on in vivo tumor growth was assessed by subcutaneously injecting PCa cells in SCID mice and intratumorally administering the medium of antiarrhythmic-treated CAFs or in co-injection experiments, where antiarrhythmic-treated CAFs were co-injected with PCa cells.

**Results:**

Activated fibroblasts show increased membrane conductance for potassium, sodium and calcium, consistently with the mRNA and protein content analysis. Antiarrhythmics modulate the expression of fibroblast activation markers. Although to a variable extent, these drugs also reduce CAF motility and hinder their ability to remodel the extracellular matrix, for example by reducing MMP-2 release. Furthermore, conditioned medium and co-culture experiments showed that antiarrhythmics can, at least in part, reverse the protumor effects exerted by CAFs on PCa cell growth and plasticity, both in androgen-sensitive and castration-resistant cell lines. Consistently, the transcriptome of antiarrhythmic-treated CAFs resembles that of tumor-suppressive NPFs. In vivo experiments confirmed that the conditioned medium or the direct coinjection of antiarrhythmic-treated CAFs reduced the tumor growth rate of PCa xenografts.

**Conclusions:**

Collectively, such data suggest a new therapeutic strategy for PCa based on the repositioning of antiarrhythmic drugs with the aim of normalizing CAF phenotype and creating a less permissive tumor microenvironment.

**Supplementary Information:**

The online version contains supplementary material available at 10.1186/s13046-024-03081-0.

## Introduction

Favorable interactions between cancer cells and the surrounding microenvironment are crucial for sustaining tumor mass formation and progression. The acquisition of malignant features, including the development of resistance to standard therapies and the enhancement of metastatic potential, is also greatly promoted by a supportive tumor microenvironment [1, 2]. Cancer-associated fibroblasts (CAFs) are the most representative non-tumorigenic cells within the tumor microenvironment. They are a highly heterogenous cellular population, in terms of phenotype and function, resulting from the cancer cell-mediated reprogramming of stroma, through the direct influence of tumor-derived cytokines and growth factors [3, 4]. Little is known about the different tumor supporting-CAF subpopulations that can cohabitate within the tumor mass, which may include immune CAFs, desmoplastic and contractile CAFs, and aggressive and secretory CAFs [5]. They are typically identified by the overexpression of activation markers, such as α-smooth muscle actin (α-SMA), fibroblast activation protein (FAP), collagens or fibroblast specific protein 1 [6], and by an increased migratory and extracellular matrix (ECM) remodeling capability [7].

Since CAFs are critically involved in cancer biology, several efforts have been recently made in the attempt to develop therapeutic strategies able to eradicate cancer-promoting CAFs from the tumor mass (reviewed in [8]). However, most of them have failed to substantially improve in cancer treatment, mainly due to the extreme complexity of this population and the lacking of unequivocal markers. Therefore, the newest therapeutic approaches in this regard are focused on restoring a suppressive phenotype rather than depleting CAFs from the tumor microenvironment [8].

As for many solid tumors, prostate cancer (PCa) stroma is characterized by a heterogenous population of CAFs with tumor-supporting features [9]. CAFs can promote proliferation, migration and invasion of PCa cells. By overproducing ECM components, such as collagens, periostin and fibronectin, and by dysregulating matrix metalloproteases proteins (e.g., MMP-2 and MMP-9), CAFs can provide physical support to PCa cells and increase matrix stiffness and remodeling, thus ultimately promoting cancer cell migration and invasion [10, 11]. CAFs can also sustain PCa progression by providing metabolic resources required for cancer cell growth. Driven by PCa cells, CAFs undergo aerobic glycolysis to release pyruvate and lactate that are taken up by cancer cells to activate mitochondrial oxidative metabolism and promote cell growth even in nutrient- or oxygen-deprived microenvironment [12]. In addition, several in vitro and in vivo studies have demonstrated that CAFs can promote the acquisition of the castration-resistant phenotype, an aggressive trait of PCa, and accelerate metastatic dissemination [13–17].

With the aim of elucidating the molecular mechanisms governing the activation of PCa stroma, we previously showed that tumor-derived IL-6 and TGF-β can both convert normal prostate fibroblasts (NPF) into CAFs with tumor-promoting features [18], confirming the complexity of tumor stroma composition in PCa [4, 18, 19]. Moreover, gene expression profiles of patient-derived CAFs and matched NPFs revealed the up-modulation of gene sets related to cardiomyopathy and cation channel activity, including genes that encode for sodium, calcium and potassium ion channels [18]. These proteins are crucial regulators of specific physiological processes, such as muscle contractions and nerve impulses, as well as the main biological processes that are frequently deregulated in cancer cells, and also in CAFs, including cell cycle progression and survival, cell migration and invasion [20–22]. Therefore, given this possible similarity between prostate CAFs and contractile cells, we set out to investigate the potential of cation channel blockers, such as antiarrhythmics, to revert CAF-activated state into a tumor suppressive phenotype. To that end, we tested class I (sodium-channel blocker, such as flecainide), class III (potassium-channel blocker, such as amiodarone) and class IV (calcium-channel blockers, such as verapamil and nifedipine) antiarrhythmics on prostate CAFs and investigated the impact of these treatments on CAF activated state and PCa-CAF interplay.

## Methods

### Cell culture

Primary CAFs and NPFs cultures were isolated from surgical specimens of three PCa-bearing patients (Gleason score 4 + 5) who underwent radical prostatectomy. Specimens were collected upon informed consent, and the study was approved by the Ethics committee of IRCCS Istituto Nazionale dei Tumori of Milano (INT n. 154/16). Intra-tumor areas or non-tumor regions of radical prostatectomy specimens were identified by an expert uro-pathologist (M.C.), selected and digested overnight at 37 °C and 5% CO_2_ in DMEM medium (Lonza, Basel, Switzerland) supplemented with 300 units/ml collagenase and 100 units/ml hyaluronidase solution (Stemcell Technologies Vancouver, Canada), 1% penicillin–streptomycin (Lonza, Basel, Switzerland) and 2.5 μg/ml of Amphotericin B. The cell suspension was centrifuged at 1,500 × g for 5 min. The resulting fibroblast-rich pellet was suspended and plated in DMEM medium (Lonza) containing 10% FBS (Thermo Fisher Scientific Inc., Waltham, MA, US), 4 mM L-glutamine and 1% penicillin–streptomycin (Lonza). CAFs or NPFs were maintained in culture for 3 passages and the absence of epithelial markers expression was verified before being used in the experiments. All established primary cultures negative for epithelial markers and expressing fibroblast markers were used until the 15th passage and maintained in DMEM medium (Lonza) containing 10% FBS (Thermo Fisher Scientific Inc.) and 4 mM L-glutamine (Lonza). PCa cell lines (DU145, PC3 and LNCaP) and the prostate myofibroblast cell line WPMY-1 were purchased from American Type Tissue Culture Collection (ATCC, VA, USA). PCa cell lines were maintained in RPMI-1640 medium (Lonza) supplemented with 10% FBS (Thermo Fisher Scientific), at 37 °C and 5% CO_2_. WPMY-1 cells were maintained in DMEM (Lonza) supplemented with 10% FBS (Gibco, Thermo Fisher Scientific Inc.). All the cell lines were authenticated and periodically monitored by genetic profiling using short tandem repeat analysis AmpFISTR Identifier PCR amplification kit (Thermo Fisher Scientific Inc.).

### Conditioned medium

For indirect co-culture and in vivo experiments, conditioned medium (CM) was collected from NPFs, antiarrhythmic-treated or untreated CAFs and DU145 cells. To obtain CM, a total of 7 × 10^5^ cells were seeded in T-75 cm^2^ culture flask. CAFs were treated with 2.5 μM of amiodarone (Hikma Pharmaceuticals, London, UK), 2.5 μM of verapamil (Abbott Laboratories, Chicago, US), 2.5 μM of nifedipine (Meda AB, Solna, Sweden) or 2.5 μM flecainide (Meda AB) for 24 h. Upon treatment, the culture medium was removed, cells were washed 2 times with PBS (Lonza) and 6 ml of serum-free medium was added for starvation. Twenty-four hours later, the CM was collected, clarified for 5 min at 1,500 × g and used freshly to treat PCa cells (DU145, PC-3 or LNCaP), or to activate NPFs or WPMY-1 or concentrated for western blotting analysis.

### Electrophysiological recordings and analyses

Whole cell patch-clamp recordings were performed on activated and control WPMY-1 fibroblasts using an Axopatch 200B amplifier (Molecular Devices) and data were sampled with a Digidata -1440 (Molecular Devices) interface (sampling time = 250 ms for voltage clamp recordings). Patch pipettes were pulled from borosilicate capillaries (Hingelberg, Malsfeld, Germany) and had 3–5 MΩ resistance before a seal was formed. Cells were recorded in a bath solution containing (in mM): NaCl 140, KCl 5, Hepes 10, glucose 5, CaCl2 2, MgCl2 1, pH 7.4. The filling solution contained (in mM): KCl 135, NaCl 10, Hepes 10, MgCl2 1, EGTA 1, CaCl2 0.1 and GTP 0.1. The pH was adjusted to 7.2 with KOH. Membrane passive properties were recorded in voltage clamp configuration. A hyperpolarizing step from -70 mV to -80 mV, normally used for evaluating the passive properties of the recorded cell [23], elicited an inward transient current used to estimate the series resistance, input resistance and membrane capacitance. Series resistance and input resistance were monitored throughout each experiment. Cells were rejected if these parameters deviated by more than 20% from the beginning of the recording. Outward currents were measured in voltage clamp by applying subsequent voltage steps of + 20 mV from a holding potential of -60 mV up to + 140 mV. Sustained outward currents (K^+^ steady) were recorded as an average of the last 15 ms of each voltage step. Transient outward currents (K + inactivated) were calculated by subtracting the sustained outward current from peak outward currents. The amplitude of the transient currents measured at the membrane potential of + 140 mV was used to report the current density. Transient inward currents (inward) were calculated as the peak outward current for each applied voltage step. For the depolarizing protocols, the PN leak subtraction of the Clampex program was used to eliminate the effects of the leakage current on the whole-cell responses [24]. The extracellular medium containing flecainide or nifedipine was bath perfused using a peristaltic pump. Pipette capacitance was compensated, and the bridge was balanced during each recording. All data were reported as the mean ± standard error of the mean (SEM). Each protocol was averaged digitally 5 times before being analyzed. All the recorded data were analyzed off-line with pCLAMP10.7 (Axon Instruments).

### Cell growth assay

PCa cells (DU145, PC-3 or LNCaP) were seeded in 12-well plates (2 × 10^4^ cells/well) and after 24 h were exposed to CM of NPFs, CM of CAFs or CM of CAF-treated with antiarrhythmics (as described above). Upon starvation with appropriate CM, cells were harvested with Trypsin–EDTA (Lonza) and counted with an automated cell counter (Beckman, Coulter, Brea, CA, US).

### Migration assay

Cell were seeded at 4 × 10^4^ cells/well into a 12-well culture plate. After 2 days, the monolayer of cells was wounded by manual scratching with a pipet tip, washed with PBS 1 × (Lonza), photographed (t0 point) and media were replaced with serum-free DMEM containing 2.5 μM of amiodarone (Hikma Pharmaceuticals), 2.5 μM of verapamil (Abbott Laboratories), 2.5 μM nifedipine (Meda AB), or 2.5 μM flecainide (Meda AB) for CAF migration experiments experiments or with CM of CAFs treated or not with antiarrhythmics (as described above) for DU145 cell migration experiments. Images of cell movement were captured at regular time intervals until 48 h by using EVOS XL – Core microscope system (Thermo Fischer Scientific Inc.).

### 3D gel collagen contraction assay

Type I collagen from rat-tail (Sigma-Aldrich, St. Louis, MO, US) was dissolved at 2 mg/ml in 0.1% acetic acid to create a stock solution. The collagen matrix was quickly prepared on ice by adding 6 ml of collagen stock solution to 3.6 ml of 0.1% acetic acid, 1.2 ml of 10 × concentrated DMEM, and 1.2 ml of sodium bicarbonate solution (11.76 mg/ml) for a final concentration of 1 mg/ml collagen. The pH was adjusted to 7.2–7.4 by adding 0.1 mol/l NaOH. CAFs or NPFs cells were then added to achieve a final concentration of 5 × 10^5^ cells/ml; gel-cells suspension was aliquoted into each well of a 24-well culture plate. After polymerization for 30 min at 37 °C, the gel in each well was overlayed with 500 μl of complete growth medium. Twenty-four hours later, CAFs were treated with 2.5 μM amiodarone (Hikma Pharmaceuticals), 2.5 μM verapamil (Abbott Laboratories), 2.5 μM nifedipine (Meda AB) or 2.5 μM flecainide (Meda AB), in serum free medium. Then, the gels were mechanically released from the wall and bottom of the wells with a sterile spatula. Gel contraction was monitored for 48 h and scanned by standardized photography at time 0 and at sequential time points.

### Gene expression profile

After RNA quality check, transcriptomic profiles of CAFs, treated or not with nifedipine (2.5 µM) or flecainide (2.5 µM), and NPFs were assessed using the Clariom™ S Human Microarray (Thermo Fisher Scientific Inc.). Raw data were normalized according to the Robust multiarray averaging (RMA) algorithm, implemented into the oligo package [25]. Normalized data were filtered removing probes with no associated official gene symbol; for probes mapping on the same gene symbol, the one with highest variance was selected. In addition, an empirical Bayes approach was applied to adjust gene expression for batch effect, using ComBat function implemented into the sva package [26]. To verify the efficacy of these analyses, the t-distributed Stochastic Neighbor Embedded (t-SNE) statistical method was employed to visualized data in a low-dimensional space since it adopts a non-linear reduction of high-dimensional transcriptomic data maintaining the similarity among samples. Differential expression analysis was performed applying a linear model implemented into the limma package [27]. A pre-ranked Gene Set Enrichment Analysis (GSEA) was performed on gene sets of the Molecular Signature Database (MSigDB), selecting Reactome pathway in the C2 collection [28]. Ranking was defined according to the t-statistic and normalized enrichment score (NES) was calculated using the functions implemented into the fgsea package [29].

Three different normalized datasets (GSE68164, GSE85606, and GSE86256) were retrieved from the Gene Expression Omnibus database [30] and for each, a pre-ranked GSEA analysis was conducted on custom selected gene sets of MSigDB, using “ion channel activity” as query in the C5 collection and using the t-statistics, obtained by linear model implemented into the limma package, as the measure for ranking gene expression in the comparison CAF vs NPF samples. A FDR threshold of 0.05 was applied to assess significant enrichments.

### Cell viability

The cytotoxic effect of amiodarone (Hikma Pharmaceuticals), verapamil (Abbott Laboratories), nifedipine (Meda AB) or flecainide (Meda AB) was determined by the CellTiter96® AQueous One Solution Cell Proliferation Assay (MTS) (Promega Corporation, Madison Wisconsin, USA). Cells were plated for 24 h in 96-well flat-bottomed microtiter plates at a density of 1.5 × 10^3^/50 μl, and then treated with increasing concentrations of amiodarone (Hikma Pharmaceuticals), verapamil (Abbott Laboratories), nifedipine (Meda AB) or flecainide (Meda AB) (0–5 µM) for 72 h. At the end of the treatment, MTS solution was added to each well and the plate was incubated for 3 h in a 5% CO_2_ incubator at 37 °C. The absorbance at 490 nm was recorded using the POLARstar optima plate-reader (VWR International, Radnor, Pennsylvania, USA).

### Immunofluorescence

CAFs were seeded at 6 × 10^4^ cell/well in a 6-well plate containing a coverslip suitable for microscopy. After 24 h, CAFs were treated with 2.5 μM amiodarone (Hikma Pharmaceuticals), 2.5 μM verapamil (Abbott Laboratories), 2.5 μM nifedipine (Meda AB) or 2.5 μM flecainide (Meda AB), in serum free medium. Twenty-four hours later cells were fixed in 4% formaldehyde dissolved in PBS for 10 min. Cells were permeabilized with cold 70% of ethanol and probed with primary antibodies for α-SMA (1:200, A2547 Sigma-Aldrich), Collagen I (1:200 ab34710; Abcam) and phospho-FAK (1:200 Y397 ab4803; Abcam) diluted in antibody diluent (S080983, Dako, Agilent Technologies) for 1 h at room temperature. Alexa Fluor594/488-labeled secondary antibody (Thermo Fisher Scientific Inc.) was used to incubate cells for 1 h at room temperature. Actin filaments were stained using phalloidin-conjugate-Fluor488 dye and nuclei were stained with DAPI (Invitrogen, Thermo Fisher Scientific Inc.). Images were acquired by Nikon Eclipse E600 microscope using ACT-1 software (Nikon, Minato City, Tokyo, Japan).

### Proteome profiler array

Proteome profiling was performed using Proteome Profiler Human Cytokine Array Kit (ARY005B, R&D, Minneapolis, MN, US) according to the manufacturer’s instructions. The array was performed on 500 μl of fivefold concentrated CM from NPFs, CAFs treated or not with nifedipine or flecainide. Chemiluminescence signals were detected using Chemi Reagent Mix provided by the kit. Semi-quantitative analysis was performed using ImageJ software (National Institutes of Health, Bethesda, MD, USA).

### In vivo experiments

Animal studies were performed in accordance with guidelines of animal care protocols approved by Ethical Committee for animal experimentation of IRCCS Istituto Nazionale dei Tumori of Milano and Italian Ministry of Health (approval code n. 350/2017-PR). Male SCID mice were purchased from Charles River Laboratories. PCa xenografts were generated by subcutaneous injection of 1 × 10^7^ DU145 cells into the right flank of SCID mice. When tumor burden reached ~ 100 mm^3^, mice were randomly assigned to control or treatment groups (*n* = 6 mice per group). For conditioned medium experiments mice were intratumorally treated (5 consecutive days for 2 weeks) with 250 μl of CM of NPFs, CAFs exposed or not to 2.5 μM amiodarone (Hikma Pharmaceuticals), 2.5 μM verapamil (Abbott Laboratories), 2.5 μM nifedipine (Meda AB) or 2.5 μM flecainide (Meda AB). At two different time points (after 1 week and at the end of the treatments) tumors were harvested. For co-injection experiments, 1 × 10^7^ DU145 cells were co-injected with NPFs or CAFs treated or not with flecainide at a ratio of 1:3 into in the right flank of SCID mice. Tumor size was measured twice a week with a Vernier caliper, and the volume was calculated using the standard modified formula: Volume (mm^3^) = (length × height^2^)/2.

### Ki-67 and CD31 staining

At the end of the treatment, mice were scarified and subcutaneous tumors were harvested, and formalix-fixed and paraffin-embedded. Tumor sections were then deparaffinised in xylene, rehydrated through graded alcohols to water, and subjected to immunohistochemical analysis using Ki-67 antibody (MIB-1, Dako; 1:200) or CD31 (MEC 13.3, sc-18916, Santa Cruz; 1:100, incubation over-night). Nuclei were counterstained with hematoxylin. Images were acquired by Nikon Eclipse E600 microscope using ACT-1 software (Nikon). At least 10 fields were scanned and the average number of Ki-67-positive or CD31-positive and negative cells was plotted.

### Statistical analysis

Statistical analysis was performed with Mann–Whitney test and Student’s t-test, when appropriate, using GraphPad Prism software (version 9.4; GraphPad Prism Inc., San Diego, CA, USA). *P* ≤ 0.05 was considered statistically significant.

### Additional methods

RNA extraction, RT-qPCR, protein isolation and western blotting protocol are described in detail in Additional Methods.

## Results

### Cation channels are up-modulated in CAFs and involved in fibroblast activation

**Fig. 1:**
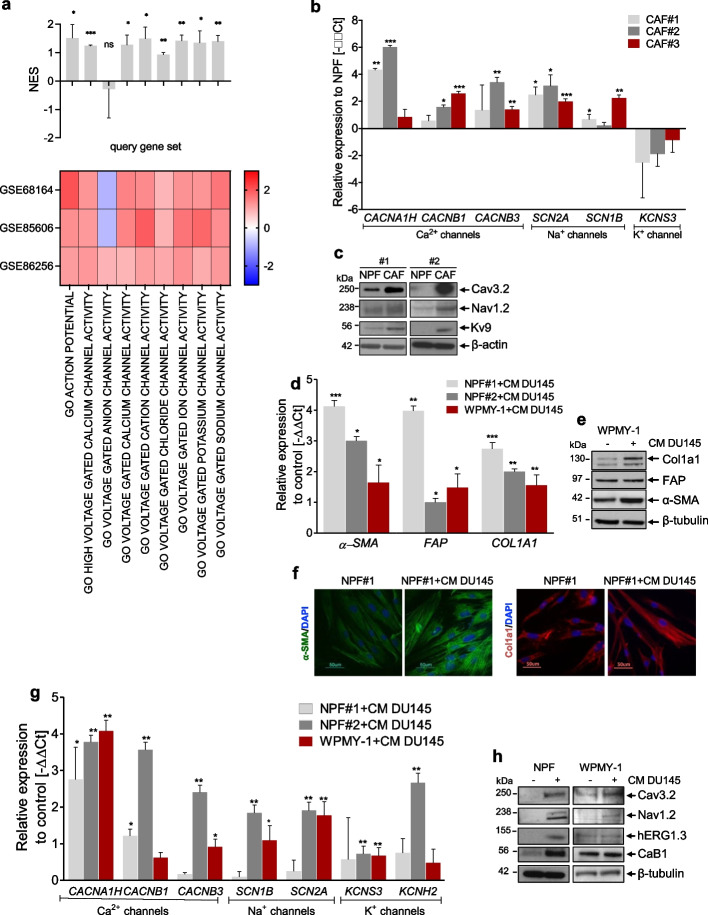
Cation channels are up-modulated in CAFs and involved in fibroblast activation. **a** Heatmap (bottom) reporting normalized enrichment scores (NES) for gene sets related to voltage gated channels and bar plot (top) reporting mean + sd, as calculated by GSEA on three independent datasets of prostate cancer patient-derived CAFs vs. matched NPFs. **b** qRT-PCR showing *CACNA1H*, *CACNB1*, *CACNB3*, *SCN2A*, *SCN1B*, and *KCNS3* expression levels in an independent setting of three CAF and matched NPF cultures. Data were reported as relative expression compared to NPF and were representative of three independent experiments. **c** Western blotting analysis showing selected ion channel protein levels in a pair of matched CAFs and NPFs. β-actin was used as endogenous control. **d** qRT-PCR indicating relative expression levels of α-SMA, FAP and COL1A1 in NPF#1, NPF#2 and WPMY-1 fibroblasts exposed to CM of DU145 cells with respect to control fibroblasts. **e** Western blotting showing α-SMA, FAP and COL1A1 expression levels in WPMY-1 fibroblasts exposed or not to CM of DU145 cells. β-tubulin was used as endogenous control. **f** Immunofluorescence microphotographs showing α-SMA (green) and Col1a1 (red) expression in NPF and NPF exposed to CM of DU145 cells. Nuclei counterstained with DAPI (blue). Scale bar, 50 µm. **g** Cation channel mRNA expression levels in NPF#1, NPF#2 and WPMY-1 fibroblasts exposed to CM of DU145 cells with respect to untreated fibroblasts. **h** Western blotting displaying cation channel protein levels in NPF#1 and WPMY-1 fibroblasts exposed or not to CM of DU145 cells. β-tubulin was used as endogenous control. Results reported in the figure represent the mean (+ SD) of three independent experiments. *p < 0.05, **p < 0.01, ***p < 0.005, Student’s t-test

Interrogation of independent gene expression data of prostate CAFs and matched NPFs confirmed our previous observation [18] showing the up-modulation of voltage-gated cation channel gene sets in CAFs (Fig. 1a). Accordingly, in an additional set of three paired cultures of CAFs and NPFs established from radical prostatectomies in our laboratory, we appreciated the up-modulation of a selected panel of cation channels, including calcium (*CACNA1H, CACNB1, CACNB3*), sodium (*SCAN2A* and *SCN1B*) and potassium channels (*KCNS3*) in CAFs compared to NPFs at either the mRNA or protein levels, or both (Fig. 1b and c, Table 1). Discrepancy observed between mRNA and protein levels of the *KCNS3*/Kv9 channel may be attributed to various post-transcriptional [31] or post-translational [32] and compensatory mechanisms that may affect protein stability, localization, or activity. As an experimental validation, we induced the “*in-vitro* activation” of patient-derived NPFs and of WPMY-1 cells (a normal prostate myofibroblast cell line) via direct exposition to conditioned medium (CM) of PCa cells (DU145). During activation, which was confirmed by the up-modulation of specific fibroblast activation markers (Fig. 1d-f), we observed the concomitant up-modulation of cation channels, including *CACNA1H*, *CACNB1*, *CACNB3*, *SCN2A*, *SCN1B*, *KCNS*3 and *KCNH2*, at both mRNA and protein levels (Fig. 1g and h). Taken together, these findings suggest the involvement of cation channels in CAF activation.

**Table 1:** List of the leading-edge genes over-expressed in prostate CAFs, corresponding encoded protein, regulated cation and antiarrhythmic drugs used to block them

Gene	Protein	Cation	Antiarrhythmic
*CACNA1H*	Cav.3.2	Ca^2+^	verapamil nifedipine
*CACNB1*	CaB1		
*CACNB2*	CaB2		
*CACNB3*	CaB3		
*KCNS3*	Kv9	K^+^	amiodarone
*KCNH2*	hERG1.3		
*KCNQ2*	Kv7.2		
*SCN1B*	NaB1	Na^+^	flecainide
*SCN2A*	Nav1.2		

### Activated fibroblasts show increased membrane conductance for potassium, sodium and calcium

**Fig. 2:**
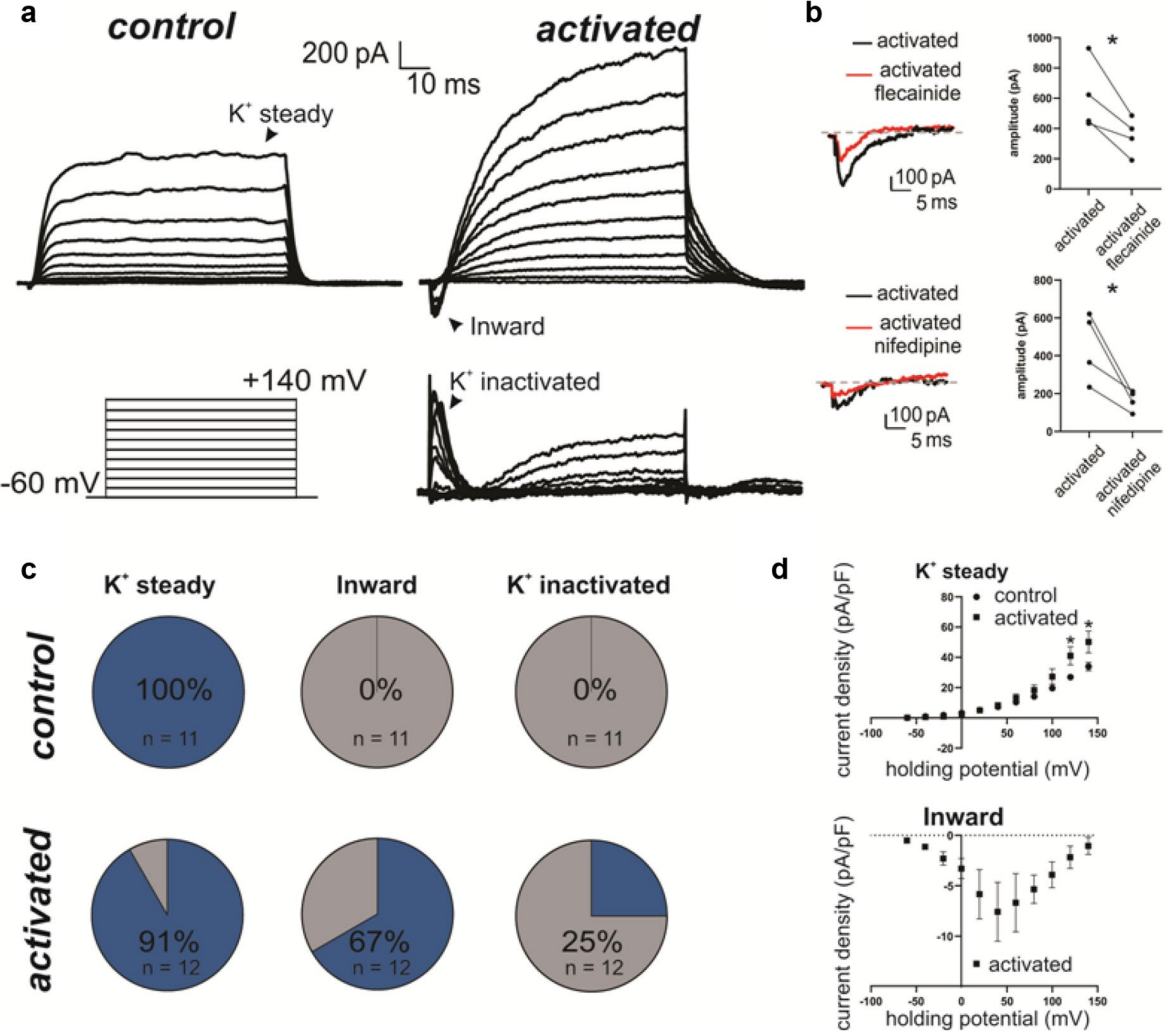
Activated fibroblasts show increased membrane conductance for potassium, sodium and calcium. **a** Representative Voltage-clamp recordings of inward and outward currents from activated and control WPMY-1 cells fibroblasts. A schematic of the applied protocol is shown in the insert. Upon activation, an inward current as well as a fast-inactivating outward current can be detected in fibroblasts. **b** The inward current shows significant sensitivity to both flecainide (2.5 μM) and nifedipine (2.5 μM) when applied to the bath solution. **c** Pie chart summarizing the percentage of cells expressing the main currents detected in A upon depolarization in both the control and the activated group. **d** The IV plot for the K + steady shows a significant increase in the current density in the activated group compared to the control condition. The I-V plot for the inward current is also displayed

Electrophysiological recordings were performed on control and activated WPMY-1 fibroblasts. The two groups were not different in any of the passive properties like membrane capacitance (control: 31.4 ± 2.4 pF, *n* = 11; activated: 32.1 ± 3.8 pF; *n* = 12; *p* = 0.96, Mann–Whitney test) or membrane resistance (control: 221. ± 27 MΩ, *n* = 11; activated: 212 ± 28 MΩ; *n* = 12; *p* = 0.82, Mann–Whitney test). In control condition, upon progressively more depolarized potentials (see methods), fibroblasts showed a very consisted current pattern in which only the sustained potassium current (K + steady) was detected (Fig. 2a). On the contrary, for activated fibroblasts we could also record an inward current (Fig. 2a) that was sensitive to both flecainide, a voltage gated sodium channel inhibitor (untreated: 609.25 ± 115.16 pA, *n* = 4; flecainide: 351.75 ± 62.16 pA; *n* = 4; *p* = 0.036, paired t-test, Fig. 2b), and nifedipine, a voltage gated calcium channel inhibitor (untreated: 449.25 ± 90.82 pA, *n* = 4; nifedipine: 164.00 ± 26.99 pA; *n* = 4; *p* = 0.037, paired t-test, Fig. 2b), suggesting that this current was mediated by influx of sodium and calcium. While the potassium steady current was present in the majority of the recorded fibroblasts in both control and activated condition (control: 100%, *n* = 11; activated: 91%, *n* = 12), the inward current as well as a fast-inactivating potassium current was functionally detected only in some of the activated fibroblasts (inward: 67%, *n* = 12; K + inactivating: 25%, *n* = 12, Fig. 2c). The current density at the peak value of the IV-plot for the K + steady current was significantly higher for the activated group compared to the control condition (control: 33.9 ± 2.8 pA/pF, *n* = 11; activated: 50.2 ± 7.2 pA/pF; *n* = 11; *p* = 0.04, Multiple unpaired t-test, Fig. 2d). The peak current density recorded for in the activated group for the K + inactivating current was 17.8 ± 7.1 pA/pF (*n* = 3) while for the inward current it was 7.6 ± 2.9 pA/pF (*n* = 8). Overall these data suggest that activated fibroblasts show increased membrane conductance for potassium, sodium and calcium, consistently with the mRNA and protein content analysis (Fig. 1).

### Antiarrhythmics counteract the activated state of prostate CAFs

**Fig. 3:**
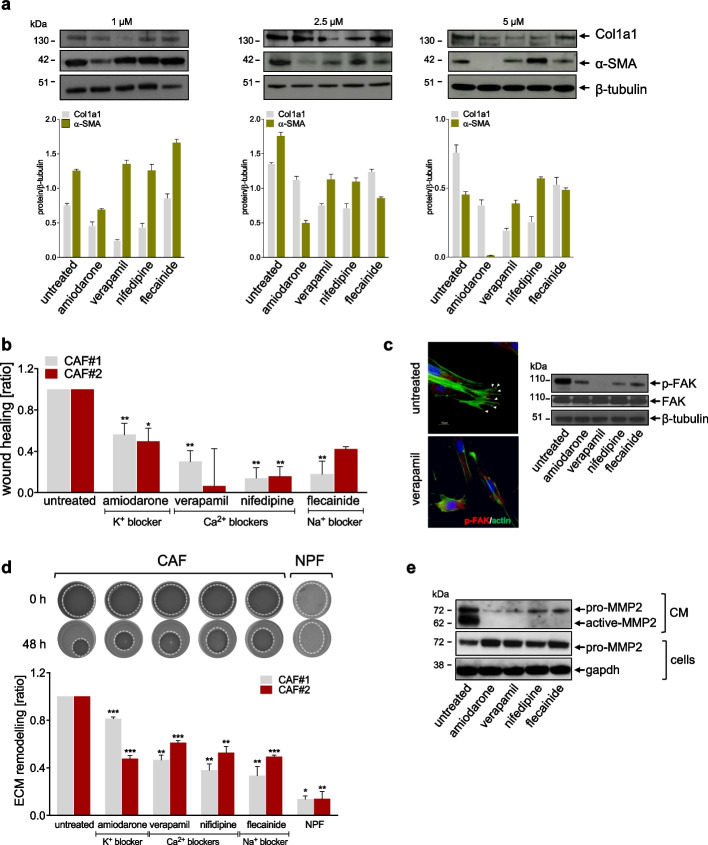
Antiarrhythmics counteract the activated state of prostate CAFs. **a** Western blotting and relative quantification showing protein levels of fibroblast activation markers (α-SMA and COL1A1) in CAFs treated for 48 h with sub-toxic doses of antiarrhythmics. β-tubulin was used as endogenous control. **b** Bar plots showing the wound-healing rate assessed by scratch assay on CAFs exposed to antiarrhythmics. Data are reported as wound healing ratio at 24 h compared to 0 h point. **c** Representative immunofluorescence microphotographs (upper panel) showing the organization of β-actin cytoskeleton (green) and p-FAK (red) in CAFs treated with verapamil as compared to untreated. Scale bar, 50 μm. Western blotting analysis (lower panel) showing p-FAK, FAK protein levels in CAFs treated with sub-toxic doses of antiarrhythmics. β-tubulin was used as endogenous control. **d** Representative images (upper panel) showing 3D-collagen gel remodeling of CAFs exposed to sub-toxic doses of antiarrhythmics. NPFs were used as negative control. The dotted lines define gel areas. Bar plots (lower panel) showing ECM remodeling ratio of treated CAFs assessed by 3D-collagen gel assay. Data are reported as ECM remodeling ratio at 48 h compared to 0 h point. **e** Western blotting showing levels of pro-MMP2 and active-MMP2 in CM from CAFs exposed or not to sub-toxic doses of antiarrhythmics and, pro-MMP2 intracellular levels in treated cells. Gapdh was used as endogenous control for cell lysate. Results reported in the figure represent the mean (+ SD) of three independent experiments. *p < 0.05, **p < 0.01, ***p < 0.005, Student’s t-test

Aiming to assess the functional role of voltage-gated cation channels in supporting fibroblast activation, a panel of pharmacological blockers of sodium, calcium and potassium channels, commonly used as antiarrhythmics, were tested as potential agents to revert CAF activated state (Table 1). The treatment of CAFs with sub-toxic doses of antiarrhythmics (Additional Fig. 1) was sufficient to induce a variable reduction of fibroblast activation markers, such as α-SMA and Col1a1 protein levels as a function of the drug and concentration (Fig. 3a). Among the several features and functions of CAFs, increased cell motility, ECM remodeling and deposition are definitely some of the main characteristics distinguishing them form NPFs [33]. The treatment with antiarrhythmics was sufficient to reduce CAF migratory ability, as indicated by the significantly reduced wound closure upon treatment (Fig. 3b and Additional Fig. 2a). In addition, p-FAK, which is a crucial mediator of cell migration and spindle orientation, was reduced, although to a variable extend, upon treatment of CAFs with antiarrhythmics (Fig. 3c), confirming that such drugs impaired CAF migratory capability by hindering focal adhesion formation. CAF-mediated ECM deposition and mechanical remodeling resulted to be affected by antiarrhythmics as well. Specifically, as depicted in Fig. 3d, the degree of contraction of gels exerted by antiarrhythmics-treated CAFs was significantly reduced compared to that of untreated CAFs. In addition, the secretion of Col1a1 and fibronectin in the extracellular environment was largely abrogated upon the treatment of CAFs with antiarrhythmics (Additional Fig. 2b), indicating a reduction of CAF-induced ECM deposition. The reason behind the reduced ECM remodeling capability of treated CAFs was investigated by measuring MMP2 levels in the CM of CAFs exposed to antiarrhythmics. As shown by western blotting (Fig. 3e), CAF-released MMP2 was significantly lower in CM of treated cells, as indicated by the reduced levels of secreted active- and pro-MMP2 in the CM of CAFs treated with antiarrhythmics compared to untreated CAFs, which was paralleled by an increased intracellular accumulation of pro-MMP2 in treated CAFs.

### Antiarrhythmics affect PCa cell growth by impairing CAF function

**Fig. 4:**
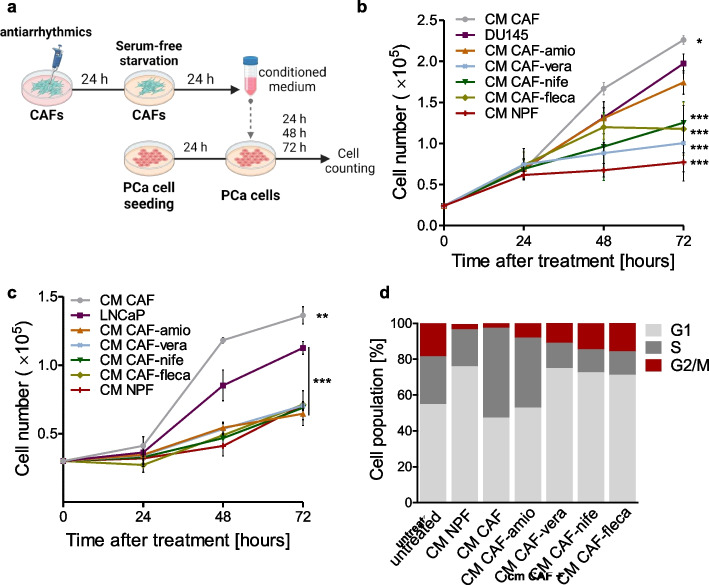
Antiarrhythmics affect PCa cell growth by impairing CAF function. **a** Schematic representation of CM experiment work-flow (Created with Biorender.com). **b** Graph reporting the growth of DU145 cells cultured with CM from CAFs treated or not with antiarrhythmics, or CM from NPFs at different time points (24, 48, 72 h). **c** Graph reporting the growth of LNCaP cells cultured with CM from CAFs treated or not with antiarrhythmics, or CM from NPFs at different time points (24, 48, 72 h). **d** Cell cycle phase distribution of DU145 cells cultured with CM from CAFs treated or not with antiarrhythmics, or CM from NPFs at 72 h until treatment. Results reported in the figure represent the mean (+ SD or ± SD) of three independent experiments. *p < 0.05, **p < 0.01, ***p < 0.005, Student’s t-test

It has been well established that CAFs induce PCa progression by supporting tumor cell growth as well as by enhancing tumor cell motility and the switch from an epithelial-like to a more mesenchymal-like phenotype [10, 34–36]. In this regard, the impact of antiarrhythmics on CAF-PCa cross-talk was evaluated by performing indirect co-culture experiments using CM (Fig. 4a). As shown in Fig. 4b,c and Additional Fig. 3a, CM of CAFs induced a slight increase of PCa cell growth, which was more pronounced in DU145 and LNCaP compared to PC3 cells [37]. In this regard, controversial information has been reported regarding the ability of CAFs to promote cell growth of PCa cell lines. In fact, high-metastatic potential PCa cell lines, like PC3 cells, were reported to be less responsive to the proliferative effects induced by CAFs compared to low-metastatic potential cell lines, like LNCaP. However, such a moderate enhancement was significantly abolished in all the PCa cell models upon exposure to CM of antiarrhythmic-treated CAFs, especially with CM-CAF-nifedipine (calcium-channel blocker) and CM-CAF-flecainide (sodium channel-blocker), partially recapitulating the tumor cell growth suppression exerted by CM of NPFs (Fig. 4b,c and Additional Fig. 3a). Consistent with cell growth findings, cell cycle analysis of DU145 cells reveled that exposure to CM of CAFs increased the S-phase cell fraction and reduced the G1-phase cell fraction compared to untreated cells, while an opposite trend was observed after exposure to CM of NPFs (Fig. 4d). CM of CAFs treated with the different antiarrhythmics, with the exception of amiodarone, was sufficient to recapitulate the effects mediated by CM-NPF on DU145 cell cycle distribution, resulting in an accumulation of cells in G1-phase and a reduction of S-phase cell population (Fig. 4d).

### Antiarrhythmics affect PCa cell plasticity by impairing CAF function

**Fig. 5:**
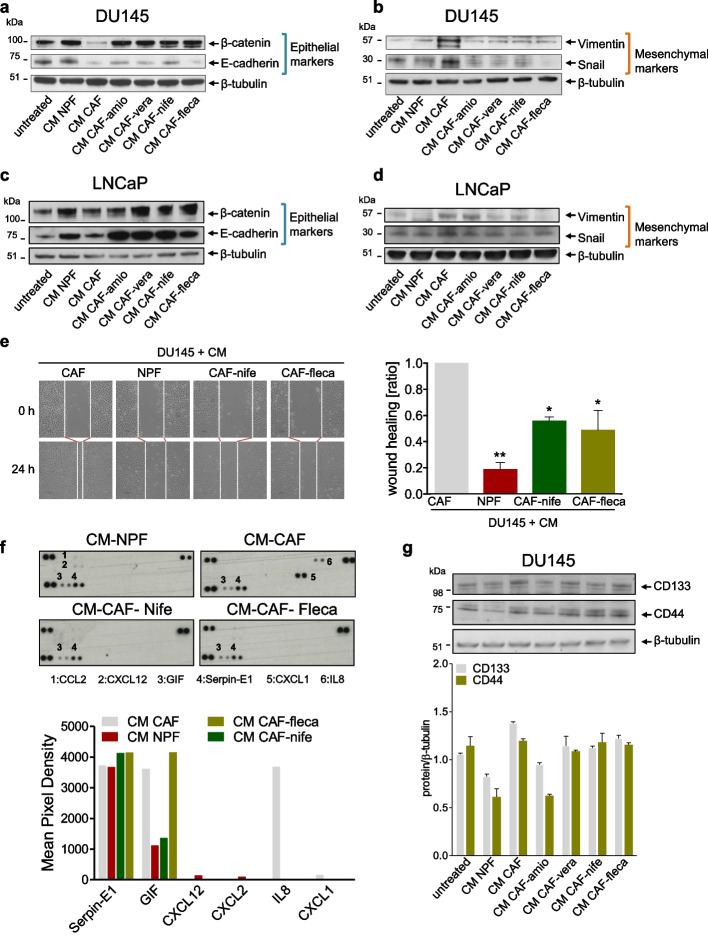
Antiarrhythmics affect PCa cell plasticity by impairing CAF function. **a**-**d** Western blotting analysis showing E-cadherin, β-catenin, Vimentin and Snail protein amount in DU145 cells (a-b) and LNCaP cells (**c**-**d**) exposed to CM from NPFs or CM from CAFs treated or not to antiarrhythmics. β-tubulin was used as endogenous control. **e** Representative bright-field microphotographs (left panel) showing migration rate of DU145 cells exposed to CM from NPFs or CM from CAFs treated or not with nifedipine or flecainide. Scale bar, 100 μm. The dotted lines define the areas lacking cells. Bar plots (right panel) showing the wound-healing rate of DU145 cells upon the indicated treatments, as from the scratch assay. Data are reported as wound healing ratio at 24 h compared to 0 h. **f** Cytokine and chemokine protein array blots (left panel) of CM from CAFs treated or not with nifedipine or flecainide, and CM from NPFs. Bar plot (right panel) depicts the pixel density of each cytokine or chemokine (mean). The signal intensity of each cytokine or chemokine was expressed relative to the mean of the intensity of the corresponding spots from vehicle control sample. **g** Western blotting and relative quantification showing the expression of stemness markers (CD133 and CD44) in DU145 cells exposed to CM from CAFs treated or not with antiarrhythmics, or CM from NPFs, with respect to untreated cells. β-tubulin was used as endogenous control. Results reported in the figure represent the mean (+ SD) of three independent experiments. *p < 0.05, **p < 0.01, ***p < 0.005, Student’s t-test., when calculated against untreated cells

As a typical CAF-induced aggressive trait, EMT markers were evaluated in the castration-resistant (DU145) and in the androgen-sensitive PCa cell model (LNCaP) exposed to CM of CAFs treated or not with antiarrhythmics. As expected, DU145 cell exposure to CM-CAF mediated a transition from a more epithelial-like toward a more mesenchymal-like phenotype, as highlighted by the down-regulation of epithelial markers (*CDH1*/E-cadherin and *CTNNB1*/β-catenin) and the increased expression of mesenchymal ones (*VIM*/vimentin and *SNAI1*/Snail) at both the mRNA and protein levels (Fig. 5a and Additional Fig. 3b). Conversely, the expression levels of *VIM*/vimentin and *SNAI1*/Snail were generally reduced in DU145 cells exposed to CM of CAF-treated with antiarrhythmics (Fig. 5b and Additional Fig. 3c), mimicking the EMT suppressive effect exerted by CM-NPF, although an enhancement of epithelial markers by CM of antiarrhythmic-treated CAFs was consistently observed only at the protein levels (Fig. 5a,b). The ability of antiarrhythmics to affect CAF-induced PCa plasticity was particularly appreciable in the androgen-sensitive model, where the treatment generally reverted the expression of both epithelial (*CDH1*/E-cadherin and *CTNNB1*/β-catenin) and mesenchymal markers (*VIM*/vimentin and *SNAI1*/Snail) in LNCaP cells, which was more appreciable at the protein levels (Fig. 5c,d and Additional Fig. 3d,e). Focusing on those antiarrhythmics that showed a greater capability to impact on ECM remodeling process mediated by CAFs (Fig. 3f) and to induce a partial reversal of EMT in co-culture experiments (Fig. 5a, b), we evaluated whether the treatment could revert CAF-promoted migratory boost on DU145 cells. As indicated in the representative photomicrographs and bar graph, DU145 cells exposed to CM of CAFs displayed a high and rapid capacity to close the wound. Conversely, the CM of CAFs treated with nifedipine or flecainide significantly reduced DU145 cell migration capability, showing a wound-healing ratio to an extent approaching that observed with CM-NPF (Fig. 5e). These findings suggest that nifedipine and flecainide decrease CAF-mediated pro-migratory boost on DU145 cells, thus confirming the repressive effects of antiarrhythmics on CAF pro-tumor spur. Since CAF-derived cytokines are master regulators of EMT, migration and invasion of cancer cells, we investigated the perturbation induced by antiarrhytmics on CAF secretome profile. Protein profiler analysis reveled an increase in the glycosylation-inhibiting factor (GIF), also known as macrophage migration inhibitory factor, in the CM form CAFs. Upon the treatment with nifedipine, GIF levels decreased in the CM of treated CAFs, bringing them closer to those observed in CM of NPFs. However, no difference in GIF levels was observed in CM of CAFs treated with flecainide compared to untreated CAFs. Additionally, IL-8, which is a well–known promoter of migration and EMT, was found to be increased in CM from CAFs [38]. In contrast, both nifedipine and flecainide completely abrogated IL-8 release from CAFs, resembling the IL-8 levels observed in the CM of NPFs (Fig. 5f). These observations indicate that antiarrhythmics perturb CAF protumor effects by reducing tumor-stroma cross-talk, potentially inhibiting the initial phases of the metastatic process, such as EMT and migration. Another interesting role exerted by CAFs is the promotion of stemness in PCs cells [34]. Thus, we investigated whether the treatment could revert CAF-promoted stemness in DU145 cells. As showed in Fig. 5g, DU145 cells exposed to CM from untreated CAF displayed a slight increase in the expression of both the stemness markers CD44 and CD133 compared to untreated cells. In contrast, CM from NPF reduced the expression of CD144 and CD133, suggesting a possible inhibition of stemness features in DU145 cells exerted by NPF. Interestingly, treatment with antiarrhythmics partially abolished the stemness-promoting effect of CM from CAF, which was particularly evident for CD133. However, this effect was observed for CD44 only when amiodarone was used (CM-CAF-amio).

### Antiarrhythmics normalize the transcriptome of CAFs

**Fig. 6:**
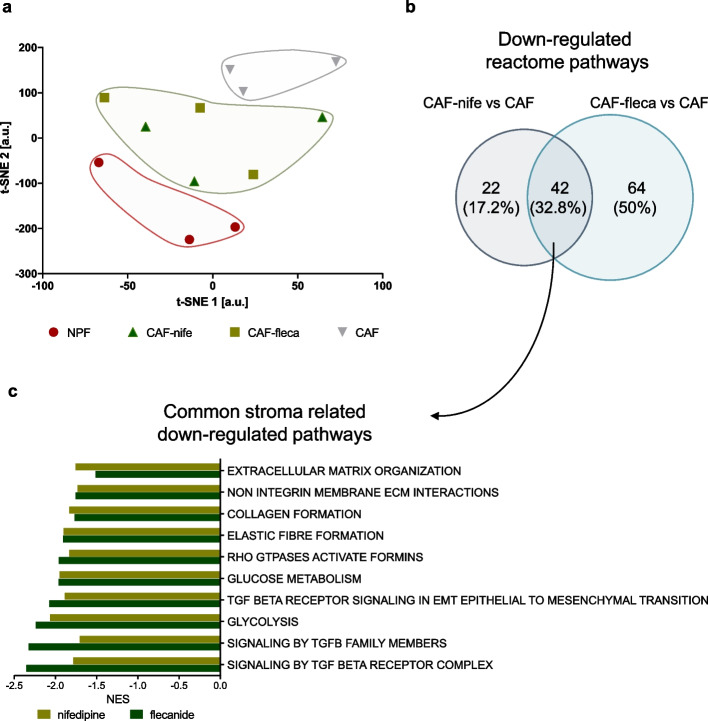
Antiarrhythmics normalize the transcriptome of CAFs. **a** Scatter plot of t-SNE components showing similarity of transcriptomes of CAFs, NPFs and antiarrhythmics-treated CAFs. **b** Venn diagram showing overlap between Reactome gene sets enriched (GSEA, NES < 0, FDR *p*-val < 0.05) in genes down-regulated in CAFs upon nifedipine and flecainide treatments. **c** Bar plot showing NES of representative gene sets down-regulated in nifedipine- and flecainide-treated CAFs

To investigate the perturbation induced by antiarrhythmics at the transcriptome level, gene expression profiling analysis was performed on three independent patient-derived CAF cultures, treated or not treated with nifedipine or flecainide, and matched NPFs as controls. The t-distributed stochastic neighborhood embedding (t-sne) projection of all genes revealed that, even though at moderate physical distance, both nifedipine and flecainide-treated CAFs clustered in between the clearly separated NPFs and CAFs clusters (Fig. 6a). This suggests that antiarrhythmics polarize CAF transcriptome into a more NPF-like one, which is in trend with the previously shown results suggesting that conversion from a tumor supporting to a tumor suppressing phenotype. Gene set enrichment analysis (GSEA) run using Reactome pathways showed commonalities between nifedipine- and flecainide-treated CAFs (Fig. 6b and Additional Fig. 4), especially regarding the down-modulation of gene sets related to extracellular matrix organization, collagen formation, TGF-beta signaling, elastic fiber formation and glucose metabolism (Fig. 6c, Additional Table 1), all processes known to be relevant for CAF activation and function [18]. Among genes up-regulated in antiarrhythmics-treated CAFs enrichment was observed for gene sets related to lipid metabolism, an aspect that might warrant investigation in future studies (Additional Table 2).

### Antiarrhythmics impair the capability of CAFs to sustain PCa cell growth in vivo

**Fig. 7:**
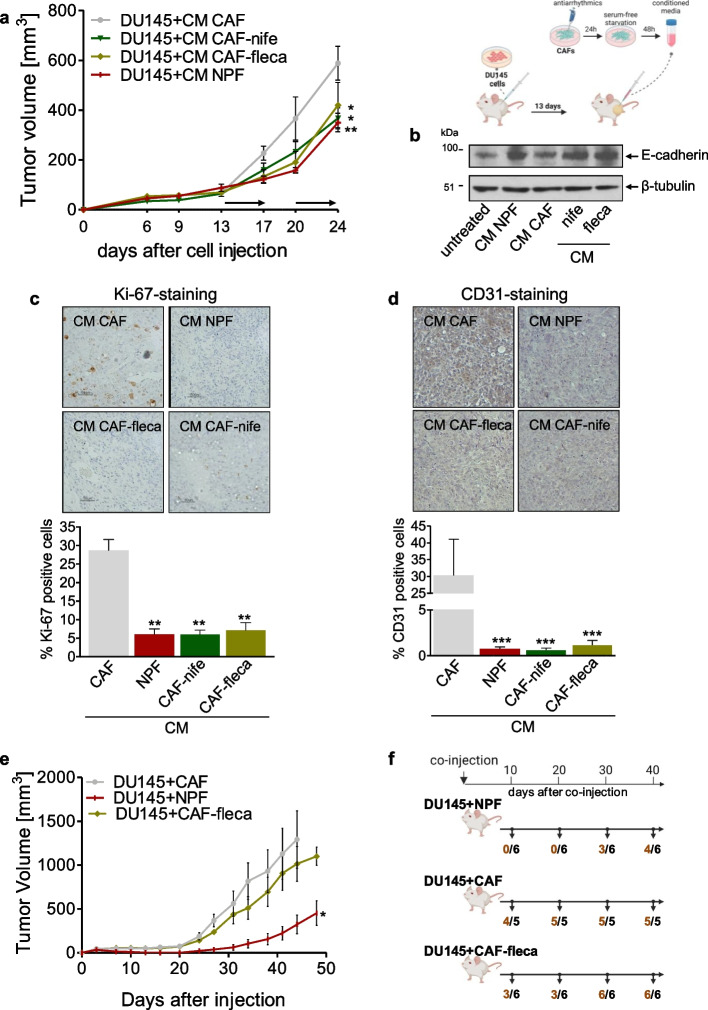
Antiarrhythmics impair the capability of CAFs to sustain PCa cell growth in vivo. **a** DU145 cells were subcutaneously injected into the right flanks of SCID mice. When tumors reached the volume of ~ 100 mm.^3^, mice were randomized into four groups and were intra-tumorally treated for 5 days per 2 weeks with CM from CAFs treated or not with nifedipine or flecainide, or CM from NPFs (see insert on the right for a schematic representation of the experiment). The graph reports tumor volumes along the experiment. Black rows indicate when treatment was administered. Schematic representation of the experimental workflow (created with Biorender.com) (**b**) Western blotting showing E-cadherin levels in PCa tumors excised at the end of the treatment with CM from CAFs treated or not with antiarrhythmics, or CM from NPFs. β-tubulin was used as endogenous control. **c** Representative bright-field microphotographs (upper panel) showing Ki-67 staining in PCa tumors excised at the end of the treatment with CM from CAFs treated or not with antiarrhythmics, or CM from NPFs. Bar plot (lower panel) showing Ki-67 positive cells in PCa tumors upon the relative treatment. Data were reported as percentage of Ki-67 positive cells with respect to total number of cells. Eight fields were evaluated for each condition. **d** Representative bright-field microphotographs (upper panel) showing CD31 staining in PCa tumors excised at the end of the treatment with CM from CAFs treated or not with antiarrhythmics, or CM from NPFs. Bar plot (lower panel) showing CD31 positive cells in PCa tumors upon the relative treatment. Data were reported as percentage of CD31 positive cells with respect to total number of cells. Eight fields were evaluated for each condition. **e** DU145 cells were subcutaneously co-injected with CAFs, CAFs pretreated with nifedipine or flecainide, or with NPFs, into the right flanks of SCID mice. The graph report tumor volumes along the experiment. **f** Timeline indicating tumor take (n. of tumors/n. of co-injected mice) in the different experimental groups, as from the experiment described in panel **e**. (Created with Biorender.com)

To evaluate the impact of antiarrhythmics on CAF-PCa cross-talk in vivo, PCa xenograft-bearing mice were treated with intra-tumorally administered CM derived from antiarrhythmic-treated CAFs, CAFs or NPFs as control (Fig. 7a). In accord with the in vitro evidence, CM of nifedipine- or flecainide-treated CAFs significantly attenuated the in vivo growth of PCa tumors compared to CM-CAF, exerting a tumor suppressive effect resembling that of CM of NPF. In addition, CM-CAF-mediated EMT was evaluated in vivo, indicating that tumors exposed to antiarrhythmic-CM CAF showed a higher expression of E-cadherin, similarly to what observed in CM-NPF treated tumors (Fig. 7b). Tumors explanted from mice exposed to CM of treated CAFs were also characterized by the lowest proliferative rate, as indicated by Ki-67 index, with a 20% reduction compared to that of tumors from CM-CAF group (Fig. 7c).

Given the well-established contribution of CAFs to promote tumor angiogenesis [3], we investigated whether CM from treated CAFs was able to impair the endothelial network formation in PCa tumors. As indicated in Fig. 7d, tumors explanted from mice exposed to CM from antiarrhythmic-treated CAFs showed a lower expression of the angiogenesis marker CD31, resembling the anti-angiogenic role exerted by CM of NPF on DU145 cells. Co-injection of DU145 cells with CAFs pretreated with flecainide (DU145 + CAF-fleca) slightly reduced tumor growth compared to DU145 cells injected with untreated CAFs, although not affecting the overall tumor take (Fig. 7e). However, DU145 cells injected with flecainide-treated CAFs required a longer interval of time to develop palpable tumors in all transplanted mice compared to DU145-CAF group (6/6 animals with palpable tumors at day 30 vs 5/5 animals with palpable tumors at day 20) (Fig. 7f). In line with NPF tumor suppressive behavior, co-injection of NPFs with DU145 cells resulted in the highest tumor growth delay and lowest xenograft take (0/6 animals with palpable tumors at day 20 after co-injection).

## Discussion

Despite the numerous attempts, cancer-centric therapeutic strategies frequently fail to overcome the malignancy, also due to the presence of a tumor-supportive microenvironment that may promote therapeutic resistance and tumor relapse [8]. Cancer initiation, progression, metastatic dissemination and multi-drug resistance are processes extensively driven by CAF-cancer cell interactions [39]. Considering this crucial role of CAFs in cancer outcome, several preclinical studies have shown that blocking CAF function may be beneficial in different cancer types [8]. However, only a few clinical trials have been conducted so far using agents or strategies specifically designed to target CAFs in cancer patients, showing very limited results. In this regard, sibrotuzumab, a humanized anti-FAP monoclonal antibody was safely administrated in phase I and II clinical trials on advanced tumors with FAP^+^ stroma, although no objective tumor response was observed [40].

The poor knowledge of the key processes governing CAF biology, together with the complexity in defining univocal markers for this heterogeneous population have impaired the translation of CAF-focused strategies into clinical practice. Therefore, further efforts are needed to fully understand CAF biology and define targetable vulnerabilities. We previously highlighted a possible involvement of cation channels in CAF activation and function, showing that gene sets encoding for calcium, sodium and potassium ion channels were up-modulated in prostate cancer-derived CAFs [18]. Here, these initial findings were confirmed both at the mRNA and protein level in independent sets of prostate CAFs established from PCa surgical samples or in NPFs experimentally activated in vitro, showing also concordance with CAF-expression profiles from publicly available data sets. Moreover, the electrophysiological recordings showed an increased membrane conductance for potassium, sodium, and calcium in activated fibroblasts. The role of cation channels has been widely studied in physiological and pathological processes, including carcinogenesis. Noteworthy, the carcinogenesis process has been recognized as a certain type of “channelopathy”, due to the functional involvement of cation channels in the main distinctive features acquired by cancer cells, including unlimited proliferation, uncontrolled differentiation and apoptosis, increased cellular motility and secretion [41].

Multiple lines of evidence pointed out that ion channels have considerable biological significance in PCa development, progression and response to therapy. For instance, the voltage-gated potassium (Kv) 2.1 was found to be upmodulated in PC3 cells and involved in cell migration. Targeting Kv2.1 with stromatoxin-1 or siRNA-mediated approaches significantly inhibited the migration of PCa cells [42]. Moreover, highly selective voltage-gated sodium channel inhibitors induced the suppression of metastasis from PCa models in vivo [43]. In addition, calcium channels were found overexpressed during androgen deprivation in PCa, suggesting their involvement in the acquisition of neuroendocrine features [44]. In this regard, we have to take into consideration that PCa presentation may include very-low risk and clinically indolent tumors, which never metastasize, or high-risk and aggressive tumors characterized by a high rate of metastatization and poor response to treatments [45, 46]. The biological mechanisms underlying these different clinical behaviors are not fully understood. Interestingly, the comparison between PCa stroma from indolent and aggressive tumors revealed a prominent difference in terms of transcriptional profile. For instance, bone-remodeling and immune-suppressive signatures have been observed in high-risk PCa stroma, but not in the stroma of indolent PCa samples [47]. This piece of evidence, together with the experimental prove of the involvement of CAFs in inducing castration-resistance in PCa, suggested that the existence of aggressive traits within the stroma can promote PCa progression toward a more aggressive phenotype. On the other hand, a less tumor-permissive stroma might eventually repress aggressive features of PCa and promote indolent behaviors.

To the best of our knowledge, only a handful of reports showed the involvement of cation channels in CAF biology [48–50]. In PCa, Vancauwenberghe and colleagues recently described that the alteration of TRPA1-calcium channel in PCa stroma is sufficient to reduce resveratrol-induced apoptosis in PCa cells, highlighting how deregulated ion channels in CAFs can affect PCa response to treatments [51]. Our work illustrates that antiarrhythmics, used as cation channel blocker agents, are able to counteract the activated state of CAFs and potentially restore a tumor-suppressive phenotype. Although at a different extent as a function of the drug and concentration used, antiarrhythmics modulate the expression of CAF markers and hinder the main tumor-promoting features of reactive prostate CAFs, including motility and capability to remodel the ECM, by reducing focal adhesion formation and MMP-2 secretion. More importantly, the use of antiarrhythmics impaired the tumor-supportive role exerted by CAFs on androgen-sensitive and –castration-resistent PCa cells, reducing their ability to foster cancer cell proliferation, plasticity, stemness, and angiogenesis both in vitro and in vivo. Normalizing activated stroma or restoring a quiescent environment has recently emerged as a valid and attractive anti-cancer CAF-centered strategy [8]. In fact, instead of depleting the tumor stroma, which could also affect cellular and structural components exerting tumor suppressive functions, reprogramming the microenvironment towards to a more quiescent phenotype should create a less permissive milieu and reduce cancer growth. In this regard, we acknowledge the existence of controversial data regarding the opportunity to reprogram the tumor microenvironment in order to re-stabilize tumor-inhibiting signals. For instance, in preclinical models of pancreatic ductal adenocarcinoma (PDA), where the role of the abundant fibrotic tumor stroma has been largely investigated, it was shown that depleting CAFs by using monoclonal antibodies against CAF markers, such as FAP or α-SMA, conferred resistance to chemotherapy [52]. Conversely, inducing a transcriptional reprogramming of pancreatic stroma cells through administration of vitamin D receptor ligands was sufficient to re-establish a physiological stroma, thus reducing tumor volume and improving survival in PDA-bearing mice [53]. Similarly, our data indicated that targeting cation channels in prostate CAFs by antiarrhythmics resulted in a reduction of PCa cell growth in mice as a consequence of a transcriptional reprogramming leading to a shift from a tumor-promoting CAF-phenotype to a tumor-suppressive one. Of note, preclinical studies reported that direct exposure to antiarrhythmics is able to reduce PCa cell proliferation in vitro and in vivo [33]. Consistent with this, clinical reports suggest that long-term use of antiarrhythmics may confer a benefit by reducing the risk of developing high-grade PCa [54]. However, while there is currently no evidence indicating a reduction in PCa-specific mortality with the use of antiarrhythmics [55], an intriguing observation comes from recent research by Fairhurst C. and colleagues. Their study revealed a potential link between the treatment with antiarrhythmics against voltage-gated sodium channels and a modest improvement in cancer-specific survival. This association was identified in a retrospective cohort of cancer patients, encompassing individuals with breast, bowel, or prostate cancer [56]. The use of antiarrhythmics in the context of PCa may offer a dual benefit by concurrently targeting the tumor and its stromal counterpart, making them a promising strategy for a comprehensive therapeutic intervention in such disease. Furthermore, exploring a drug repositioning strategy has the advantage of potentially reducing the time and costs associated with developing new compounds.

## Conclusions

Here, we speculate on the use of antiarrhythmics as a potential repositioning strategy to normalize PCa stroma through the inhibition of voltage-gated cation channels. Our results show that antiarrhythmics are indeed able to modulate CAF-activated phenotype and impair the CAF-mediated pro-tumor boost on PCa cells both in vitro and in vivo.

### Supplementary Information

Additional file 1: Additional Figure 1. Dose-response curves of CAFs exposed to antiarrhythmics for 72 h. Results reported in the figure represent the mean (+SD or ±SD) of three independent experiments.

Additional file 2: Additional Figure 2. a. Representative bright-field microphotographs showing migration rate of CAFs exposed to antiarrhythmics Scale bar, 100 μm. The dotted lines define the areas lacking cells. b. Western blotting showing fibronectin and Col1a1 in CM from CAFs treated or not with antiarrhythmics.

Additional file 3: Additional Figure 3. a. Graph reporting the growth of PC3 cells cultured with CM from CAFs treated or not with antiarrhythmics, or CM from NPFs at different time points (24, 48, 72 hours). b and d. qRT-PCR showing relative expression levels of epithelial markers (*CDH1 *and *CTNNB1*) in DU145 cells (b) or LNCaP cells (d) exposed to CM from CAFs treated or not with antiarrhythmics, or CM from NPFs with respect to untreated cells. c and e. qRT-PCR showing relative expression levels of mesenchymal markers (*VIM *and *SNAI1*) in DU145 cells (c) and LNCaP cells (e) exposed to CM from CAFs treated or not with antiarrhythmics, or CM from NPFs with respect to untreated cells.

Additional file 4: Additional Figure 4. Venn diagram showing overlap between Reactome gene sets enriched (GSEA, NES<0, adjusted p-val<0.05) in genes up-regulated in CAFs upon nifedipine and flecainide treatments.

Additional file 5: Additional Table 1. Commonly down-regulated Reactome genesets in antiarrhythmics-treated CAFs.

Additional file 6: Additional Table 2. Commonly up-regulated Reactome genesets in antiarrhythmics-treated CAFs.

Additional file 7.

## Data Availability

All data generated or analyzed during this study are included in this published article (and its Additional files) and available from the corresponding author on reasonable request.

## Abbreviations

α-SMA: α-Smooth muscle actin
CAFs: Cancer-associated fibroblasts
CM: Conditioned medium
ECM: Extracellular matrix
EMT: Epithelial-mesenchymal transition
FAP: Fibroblast activation protein
FC: Fold change
GSEA: Gene Set Enrichment Analysis
MMPs: Matrix metalloproteases proteins
NES: Normalized enrichment score
NPFs: Normal prostate fibroblasts
PCa: Prostate cancer
PDA: Pancreatic ductal adenocarcinoma
MSigDB: Molecular Signature Database
Kv: Voltage-gated potassium
