# Supplementary figures and images for: Repositioning of antiarrhythmics for prostate cancer treatment: a novel strategy to reprogram cancer-associated fibroblasts towards a tumor-suppressive phenotype
Source: J Exp Clin Cancer Res. 2024 Jun 11;43:161. doi: 10.1186/s13046-024-03081-0 (PMC11165820; doi:10.1186/s13046-024-03081-0)

**a**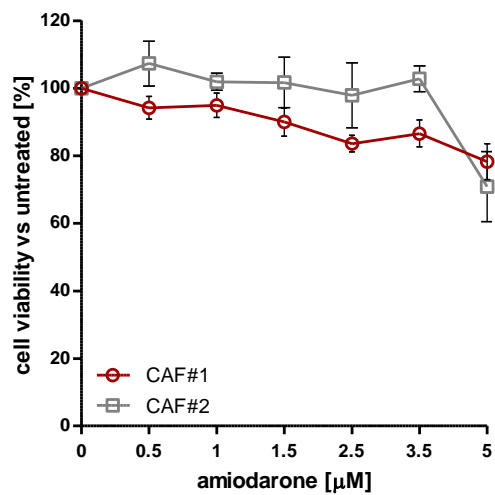**b**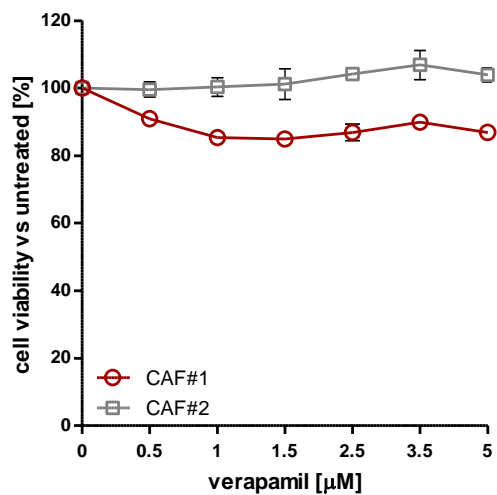**c**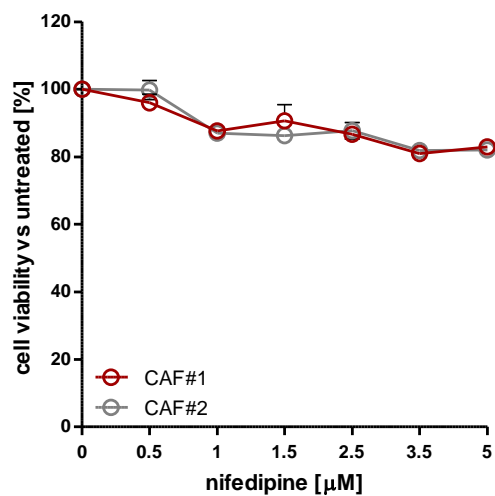**d**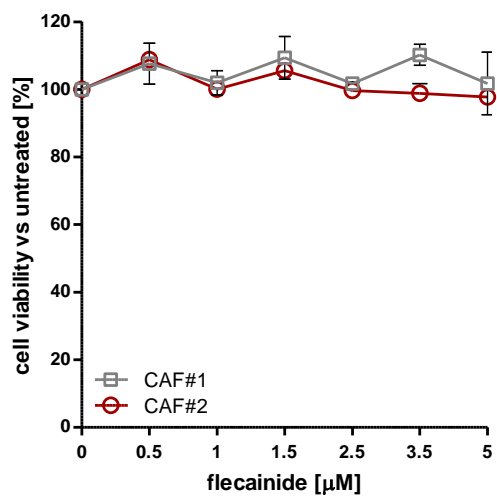**Additional Figure 1**

Supplement: Supplementary file 1 — Additional file 1: Additional Figure 1. Dose-response curves of CAFs exposed to antiarrhythmics for 72 h. Results reported in the figure represent the mean (+SD or ±SD) of three independent experiments. [file 13046_2024_3081_MOESM1_ESM.pdf]

**a**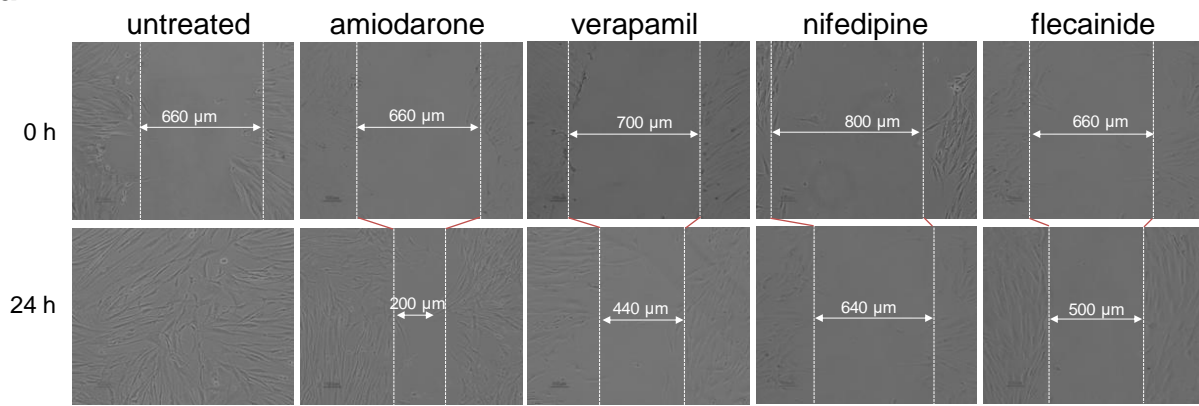**b**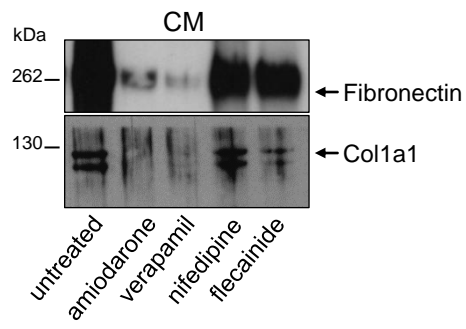**Additional Figure 2**

Supplement: Supplementary file 2 — Additional file 2: Additional Figure 2. a. Representative bright-field microphotographs showing migration rate of CAFs exposed to antiarrhythmics Scale bar, 100 μm. The dotted lines define the areas lacking cells. b. Western blotting showing fibronectin and Col1a1 in CM from CAFs treated or not with antiarrhythmics. [file 13046_2024_3081_MOESM2_ESM.pdf]

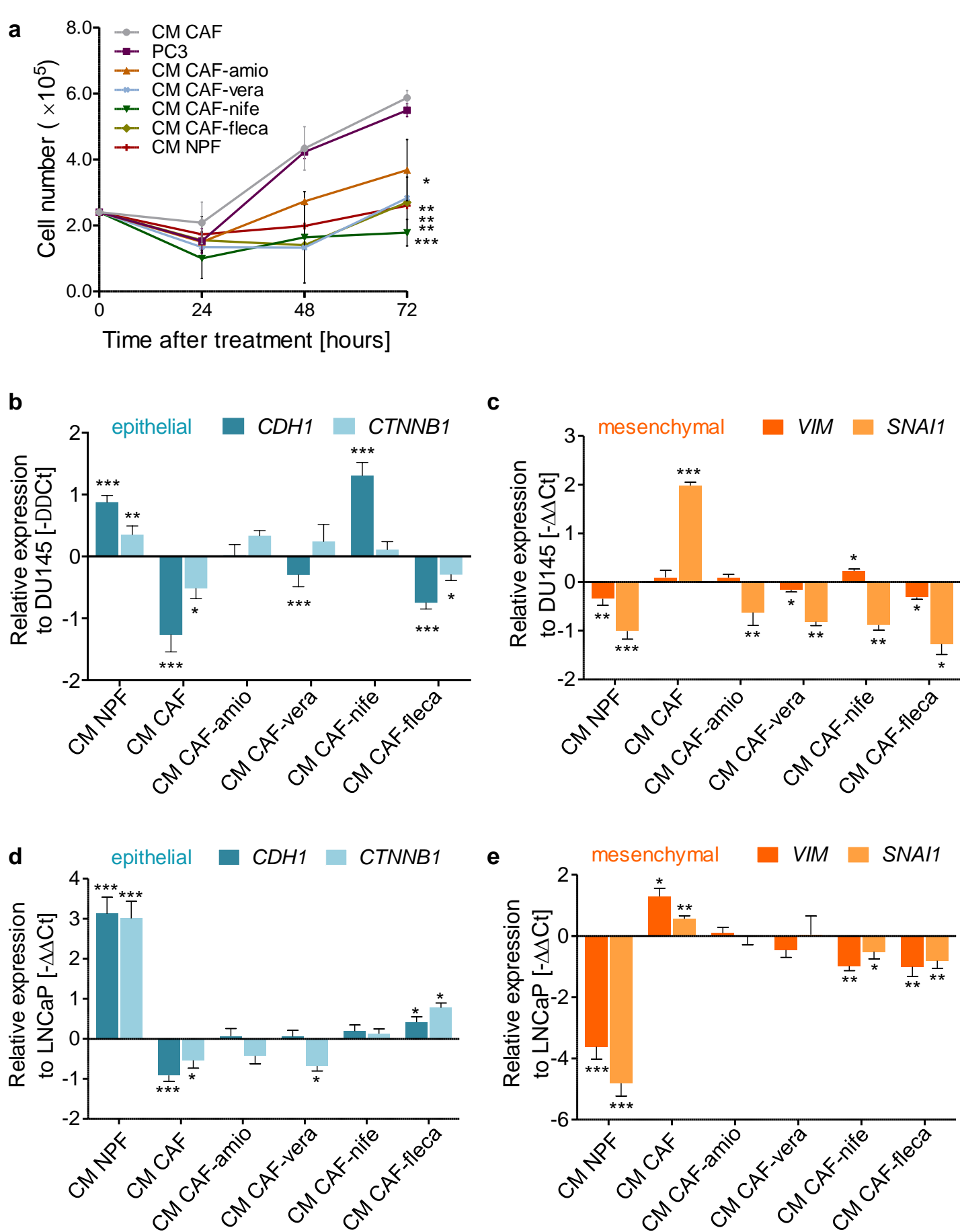

Additional Figure 3

Supplement: Supplementary file 3 — Additional file 3: Additional Figure 3. a. Graph reporting the growth of PC3 cells cultured with CM from CAFs treated or not with antiarrhythmics, or CM from NPFs at different time points (24, 48, 72 hours). b and d. qRT-PCR showing relative expression levels of epithelial markers (CDH1 and CTNNB1) in DU145 cells (b) or LNCaP cells (d) exposed to CM from CAFs treated or not with antiarrhythmics, or CM from NPFs with respect to untreated cells. c and e. qRT-PCR showing relative expression levels of mesenchymal markers (VIM and SNAI1) in DU145 cells (c) and LNCaP cells (e) exposed to CM from CAFs treated or not with antiarrhythmics, or CM from NPFs with respect to untreated cells. [file 13046_2024_3081_MOESM3_ESM.pdf]

**a**

Up-regulated  
reactome pathways

CAF-nife vs CAF

CAF-fleca vs CAF

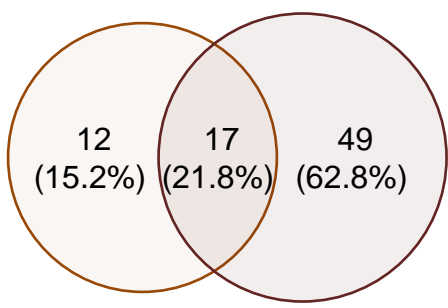

**Additional Figure 4**

Supplement: Supplementary file 4 — Additional file 4: Additional Figure 4. Venn diagram showing overlap between Reactome gene sets enriched (GSEA, NES<0, adjusted p-val<0.05) in genes up-regulated in CAFs upon nifedipine and flecainide treatments. [file 13046_2024_3081_MOESM4_ESM.pdf]
