# Supplementary material for: Repositioning of antiarrhythmics for prostate cancer treatment: a novel strategy to reprogram cancer-associated fibroblasts towards a tumor-suppressive phenotype
Source: J Exp Clin Cancer Res. 2024 Jun 11;43:161. doi: 10.1186/s13046-024-03081-0 (PMC11165820; doi:10.1186/s13046-024-03081-0)
Supplement: Supplementary file 5 — Additional file 5: Additional Table 1. Commonly down-regulated Reactome genesets in antiarrhythmics-treated CAFs. [file 13046_2024_3081_MOESM5_ESM.docx]

**Additional Table 1.** Commonly down-regulated Reactome genesets in antiarrhythmics-treated CAFs.

| **Down-regulated Reactome genesets** | **CAF-Fleca *vs***  **CAF** | | **CAF-Nife *vs***  **CAF** | |
| --- | --- | --- | --- | --- |
|  | **NES** | **FDR** | **NES** | **FDR** |
| Srp Dependent Cotranslational Protein Targeting to Membrane | -2.47 | 3.80e-08 | -2.10 | 9.48e-05 |
| Eukaryotic Translation Elongation | -2.47 | 3.76e-07 | -2.24 | 3.90e-04 |
| Signaling By TGF-β Beta Receptor Complex | -2.35 | 4.01e-06 | -1.78 | 1.51e-02 |
| Signaling By TGF-β Family Members | -2.33 | 1.53e-06 | -1.70 | 1.34e-02 |
| Glycolysis | -2.24 | 6.83e-05 | -2.06 | 4.13e-04 |
| Influenza Infection | -2.13 | 3.81e-06 | -1.66 | 8.32e-03 |
| Response of Eif2ak4 Gcn2 To Amino Acid Deficiency | -2.11 | 6.83e-05 | -1.64 | 3.08e-02 |
| Activation of ATR In Response to Replication Stress | -2.10 | 1.95e-03 | -2.25 | 3.61e-04 |
| Unfolded Protein Response UPR | -2.01 | 4.21e-04 | -1.74 | 1.03e-02 |
| HDR Through Single Strand Annealing Ssa | -2.00 | 5.57e-03 | -2.20 | 8.17e-04 |
| Processing of DNA Double Strand Break Ends | -1.98 | 2.40e-03 | -1.97 | 4.70e-03 |
| Glucose Metabolism | -1.96 | 7.44e-04 | -1.95 | 8.78e-04 |
| Resolution of Sister Chromatid Cohesion | -1.96 | 4.02e-04 | -1.91 | 1.34e-03 |
| Rho Gtpases Activate Formins | -1.96 | 1.34e-04 | -1.83 | 2.09e-03 |
| Mitotic Spindle Checkpoint | -1.96 | 2.62e-04 | -1.95 | 7.60e-04 |
| Mitotic Prometaphase | -1.94 | 2.50e-05 | -1.90 | 1.40e-04 |
| Homologous DNA Pairing and Strand Exchange | -1.91 | 6.26e-03 | -2.34 | 1.40e-04 |
| Elastic Fibre Formation | -1.91 | 9.72e-03 | -1.90 | 1.41e-02 |
| TP53 Regulates Transcription of Additional Cell Cycle Genes Whose Exact Role in The P53 Pathway Remain Uncertain | -1.84 | 2.87e-02 | -1.85 | 3.70e-02 |
| Recognition of DNA Damage by PCNA Containing Replication Complex | -1.83 | 3.39e-02 | -1.95 | 1.54e-02 |
| Asparagine N-Linked Glycosylation | -1.81 | 1.99e-05 | -1.70 | 2.69e-04 |
| ER to Golgi Anterograde Transport | -1.78 | 7.84e-04 | -1.69 | 4.34e-03 |
| Homology Directed Repair | -1.77 | 5.57e-03 | -2.04 | 2.69e-04 |
| Collagen Formation | -1.77 | 8.46e-03 | -1.80 | 4.48e-03 |
| Intra Golgi And Retrograde Golgi to ER Traffic | -1.76 | 5.54e-04 | -1.66 | 4.53e-03 |
| Non Integrin Membrane ECM Interactions | -1.76 | 2.98e-02 | -1.73 | 3.89e-02 |
| G2/M DNA Damage Checkpoint | -1.75 | 1.57e-02 | -1.87 | 8.11e-03 |
| HDR Through Homologous Recombination Hrr | -1.75 | 1.06e-02 | -1.99 | 1.30e-03 |
| Copi Mediated Anterograde Transport | -1.71 | 1.21e-02 | -1.71 | 1.62e-02 |
| Chromosome Maintenance | -1.70 | 1.63e-02 | -1.88 | 3.96e-03 |
| Rho GTPase Effectors | -1.67 | 3.82e-04 | -1.50 | 1.03e-02 |
| Transport to The Golgi And Subsequent Modification | -1.67 | 2.40e-03 | -1.25 | 1.34e-02 |
| Cell Cycle | -1.63 | 1.08e-05 | -1.57 | 9.48e-05 |
| Cell Cycle Mitotic | -1.61 | 5.18e-05 | -1.59 | 3.27e-04 |
| DNA Double Strand Break Repair | -1.59 | 1.62e-02 | -1.65 | 1.34e-02 |
| M Phase | -1.58 | 7.94e-04 | -1.48 | 1.08e-02 |
| Copi Dependent Golgi to ER Retrograde Traffic | -1.56 | 3.59e-02 | -1.66 | 4.53e-03 |
| Extracellular Matrix Organization | -1.51 | 8.17e-03 | -1.76 | 1.43e-04 |
| Cell Cycle Checkpoints | -1.50 | 5.57e-03 | -1.70 | 3.45e-04 |
| Metabolism of Carbohydrates | -1.48 | 1.16e-02 | -1.51 | 8.11e-03 |
| DNA Repair | -1.38 | 3.59e-02 | -1.49 | 1.83e-02 |
| Post Translational Protein Modification | -1.33 | 4.02e-04 | -1.25 | 1.34e-02 |
