# Supplementary material for: Repositioning of antiarrhythmics for prostate cancer treatment: a novel strategy to reprogram cancer-associated fibroblasts towards a tumor-suppressive phenotype
Source: J Exp Clin Cancer Res. 2024 Jun 11;43:161. doi: 10.1186/s13046-024-03081-0 (PMC11165820; doi:10.1186/s13046-024-03081-0)
Supplement: Supplementary file 6 — Additional file 6: Additional Table 2. Commonly up-regulated Reactome genesets in antiarrhythmics-treated CAFs. [file 13046_2024_3081_MOESM6_ESM.docx]

**Additional Table 2.** Commonly up-regulated Reactome genesets in antiarrhythmics-treated CAFs.

| **Up-regulated Reactome genesets** | **CAF-Fleca *vs***  **CAF** | | **CAF-Nife *vs***  **CAF** | |
| --- | --- | --- | --- | --- |
|  | **NES** | **FDR** | **NES** | **FDR** |
| Metabolism of Lipids | 1.29 | 3.39e-02 | 1.76 | 7.75e-08 |
| Transport of Small Molecules | 1.48 | 8.97e-04 | 1.53 | 2.69e-04 |
| Fatty Acid Metabolism | 1.57 | 1.69e-02 | 1.75 | 1.82e-03 |
| Transport of Inorganic Cations Anions and Amino Acids Oligopeptides | 1.61 | 2.26e-02 | 1.64 | 2.63e-02 |
| Metabolism of Steroids | 1.65 | 7.72e-03 | 1.67 | 1.08e-02 |
| Synthesis Of 16 20 Hydroxyeicosatetraenoic Acids Hete | 1.77 | 3.75e-02 | 2.00 | 8.11e-03 |
| Biological Oxidations | 1.77 | 4.93e-04 | 1.85 | 3.00e-04 |
| Metabolic Disorders of Biological Oxidation Enzymes | 1.81 | 2.04e-02 | 2.25 | 3.45e-04 |
| Endogenous Sterols | 1.82 | 2.17e-02 | 2.23 | 3.27e-04 |
| Pregnenolone Biosynthesis | 1.87 | 3.70e-02 | 1.87 | 2.60e-02 |
| Arachidonic Acid Metabolism | 1.88 | 9.72e-03 | 1.74 | 3.70e-02 |
| Phase I Functionalization of Compounds | 1.91 | 7.94e-04 | 2.20 | 1.71e-05 |
| Abc Family Proteins Mediated Transport | 1.92 | 9.18e-04 | 1.66 | 2.43e-02 |
| Scf Skp2 Mediated Degradation of P27 P21 | 1.96 | 3.24e-03 | 1.72 | 3.95e-02 |
| Synthesis of Bile Acids and Bile Salts Via 27 Hydroxycholesterol | 1.97 | 1.39e-02 | 1.94 | 1.70e-02 |
| Negative Regulation of Notch4 Signaling | 2.07 | 1.42e-03 | 1.85 | 1.08e-02 |
| Cytochrome P450 Arranged by Substrate Type | 2.13 | 1.78e-04 | 2.30 | 4.24e-05 |
| Transport of Small Molecules | 1.48 | 8.97e-04 | 1.53 | 2.69e-04 |
| Fatty Acid Metabolism | 1.57 | 1.69e-02 | 1.75 | 1.82e-03 |
| Transport of Inorganic Cations Anions and Amino Acids Oligopeptides | 1.61 | 2.26e-02 | 1.64 | 2.63e-02 |
| Metabolism of Steroids | 1.65 | 7.72e-03 | 1.67 | 1.08e-02 |
| Synthesis Of 16 20 Hydroxyeicosatetraenoic Acids (HETE) | 1.77 | 3.75e-02 | 2.00 | 8.11e-03 |
| Biological Oxidations | 1.77 | 4.93e-04 | 1.85 | 3.00e-04 |
| Metabolic Disorders of Biological Oxidation Enzymes | 1.81 | 2.04e-02 | 2.25 | 3.45e-04 |
