# Supplementary material for: Repositioning of antiarrhythmics for prostate cancer treatment: a novel strategy to reprogram cancer-associated fibroblasts towards a tumor-suppressive phenotype
Source: J Exp Clin Cancer Res. 2024 Jun 11;43:161. doi: 10.1186/s13046-024-03081-0 (PMC11165820; doi:10.1186/s13046-024-03081-0)
Supplement: Supplementary file 7 — Additional file 7. [file 13046_2024_3081_MOESM7_ESM.docx]

**Supplementary Materials and Methods**

***RNA isolation and RT-qPCR***

Total RNA was isolated using QIAzol Lysis Reagent and miRNeasy Mini Kit (QIAGEN, Hilden, Germany) with DNase I digestion (QIAGEN), according to the manufacturer’s instructions. RNA yield and A260/280 ratio were monitored with a NanoDrop ND-2000c spectrophotometer (Thermo Fisher Scientific Inc.). cDNA was synthesized using a high-capacity cDNA Reverse Transcription Kit (Thermo Fisher Scientific Inc.). Quantification of gene expression was assessed by RT-qPCR using No AmpErase TaqMan Universal PCR Master Mix (Thermo Fisher Scientific Inc.) and the following specific TaqMan gene expression assays (Thermo Fisher Scientific Inc.) were used: *CACNA1H* Hs01103527_m1, *CACNB1* Hs00609503_m1, *CACNB3* Hs0016787_m1, *SCN2A* Hs01109871_m1, *SCN1B* Hs00962350_m1, *KCNS3* Hs04234270_m1, *KCNH2* Hs04234675_m1, *ACTA2* Hs00909449_m1 (α-SMA), *FAP* Hs00990806_m1, *COL1A1* Hs00164004_m1, *CDH1* Hs00170423_m1, *CTNNB1* Hs00355045_m1, *VIM* Hs00185584_m1 and *SNAI1* Hs00195591_m1. For comparative analyses, *GAPDH* (PN4326317E) (Thermo Fisher Scientific Inc.) was measured as endogenous control. RT-qPCR results were reported as relative quantity (RQ = 2−ddCt) with respect to a calibrator sample, using the comparative Ct (ddCt) method.

***Protein Extraction and Western Blotting***

Cells were lysed with 1× RIPA lysis buffer supplemented with ProteaseARREST™ cocktail (G-Bioscience, St. Louis, MO US), according to the manufacturer’s instructions. Frozen PCa xenografts harvested at the end of the treatment were mechanically homogenized and then lysed with 1× RIPA lysis buffer supplemented with ProteaseARREST™ cocktail (G-Bioscience), according to the manufacturer’s instructions. For the assessment of secreted proteins, an equal number of cells was seeded, and then an equal volume of conditioned medium (CM) was collected and clarified for 10 min at 1,500×g and 5-fold concentrated using Concentrator Spin 5K MVCO column (Agilent Technologies, Santa Clara, CA, US). Twenty micrograms of lysates or 2.5 μL of 5-fold concentrated CM were resolved in 4–12% precast Bis–Tris sodium dodecyl sulfate-polyacrylamide or in 3-8% precast Tris-acetate gels (Thermo Fisher Scientific Inc.) and transferred onto Hybond nitrocellulose membranes (GE Healthcare Life Sciences, Buckinghamshire, UK). Filters were blocked for non-specific reactivity by incubation for 1 h at room temperature in 5% skim milk dissolved in 1× PBS-0.1% Tween 20 and probed overnight at 4 °C with the following antibodies: CACNAH1/Cav3.2 (1:500 ab128251; Abcam, Cambridge, UK), CACNB1/CaB1 (1:500 ab155179; Abcam) KCNS3/Kv9 (1:500 ab34710; Abcam), KCNH2/h.ERG1.3 (1:500 ab196301, Abcam), SCN2A/Na1.2 (1:500 ab65163; Abcam), α-SMA (1:3000, A2547 Sigma-Aldrich), Collagen I (1:1000 ab34710; Abcam), FAP (1:500 ab28244, Abcam) phospho-FAK (1:500 Y397 ab4803; Abcam), FAK (1:1000 ab131435; Abcam), E-cadherin (1:1000 sc-7870 H-108, Santa Cruz Biotechnology, Santa Cruz, CA, US), β-catenin (1,:500 sc59891, Santa Cruz Biotechnology), Vimentin (1:1000 sc32322, Santa Cruz Biotechnology), Snail (1.500 C15D3 #3879, Cell Signaling, Danvers, MA, US), MMP2 (1:200 ab92536; Abcam), Fibronectin (1:500 ab2413; Abcam), CD133 (1:500, D2V8Q #64326, Cell Signaling), CD44 (1:500, E7K2Y #37259, Cell Signaling). Anti-β-actin (1:5000, ab8227; Abcam) or β-tubulin (1:3000 ab6046, Abcam) or GAPDH antibody (G8795, Sigma-Aldrich) was used as a control for equal protein loading. Filters were incubated with the secondary horseradish peroxidase-conjugated anti-mouse (NA931V, GE Health-care Life Sciences) or anti-rabbit (NA9340V, GE Healthcare Life Sciences) antibodies for 1 h at room temperature. Immunoreactivity was detected by the enhanced chemiluminescence (ECL) immunodetection system (WP20005, Thermo Fisher Scientific Inc.). Membranes were cut to allow simultaneous incubation of different primary antibodies on the same samples. For the preparation of figures, we cropped the original western blot to generate the appropriate figure panels with the relevant lanes. The cropped image was then subjected to uniform image enhancement of contrast and brightness. Molecular weights were determined using SeeBlue™ Plus2 or HiMark™ Pre-stained Protein Standards (Invitrogen, Thermo Fisher Scientific).
